# In Vitro Maturation of Dopaminergic Neurons Derived from Mouse Embryonic Stem Cells: Implications for Transplantation

**DOI:** 10.1371/journal.pone.0031999

**Published:** 2012-02-22

**Authors:** Bradley Watmuff, Colin W. Pouton, John M. Haynes

**Affiliations:** Stem Cell Biology Group, Monash Institute of Pharmaceutical Sciences, Monash University, Parkville, Victoria, Australia; University of Southern California, United States of America

## Abstract

The obvious motor symptoms of Parkinson's disease result from a loss of dopaminergic neurons from the substantia nigra. Embryonic stem cell-derived neural progenitor or precursor cells, adult neurons and fetal midbrain tissue have all been used to replace dying dopaminergic neurons. Transplanted cell survival is compromised by factors relating to the new environment, for example; hypoxia, mechanical trauma and excitatory amino acid toxicity. In this study we investigate, using live-cell fluorescence Ca^2+^ and Cl^−^ imaging, the functional properties of catecholaminergic neurons as they mature. We also investigate whether GABA has the capacity to act as a neurotoxin early in the development of these neurons. From day 13 to day 21 of differentiation [Cl^−^]_i_ progressively dropped in tyrosine hydroxylase positive (TH^+^) neurons from 56.0 (95% confidence interval, 55.1, 56.9) mM to 6.9 (6.8, 7.1) mM. At days 13 and 15 TH^+^ neurons responded to GABA (30 µM) with reductions in intracellular Cl^−^ ([Cl^−^]_i_); from day 21 the majority of neurons responded to GABA (30 µM) with elevations of [Cl^−^]_i_. As [Cl^−^]_i_ reduced, the ability of GABA (30 µM) to elevate intracellular Ca^2+^ ([Ca^2+^]_i_) did also. At day 13 of differentiation a three hour exposure to GABA (30 µM) or L-glutamate (30 µM) increased the number of midbrain dopaminergic (TH^+^ and Pitx3^+^) neurons labeled with the membrane-impermeable nuclear dye TOPRO-3. By day 23 cultures were resistant to the effects of both GABA and L-glutamate. We believe that neuronal susceptibility to amino acid excitotoxicity is dependent upon neuronal maturity, and this should be considered when isolating cells for transplantation studies.

## Introduction

Embryonic stem cells (ESCs) can be propagated in culture and can differentiate into any cell type of the adult form [1]. The ability to drive these cells toward particular lineages makes them useful models for pharmacological investigations or research tools in drug discovery programs [2], [3], [4], [5]. However, much of current impact of stem cell research arises from their potential to replace or regenerate damaged tissue [6], [7]. One major ESC-derived cell transplantation target are the dopaminergic neurons of the substantia nigra that degenerate in Parkinson's disease (PD) [8], [9]. Transplantation studies aim to correct the functional deficit that becomes evident as the resident neurons die. To date, these studies have used cells at all levels of neural differentiation, from neuronal stem cells to post-mitotic Pitx3-expressing neurons [8], [9], [10], [11], [12], [13], [14], [15]. One problem that continues to plague transplantation therapies is the low survival rate of transplanted neurons [16], [17], [18], [19]. This is not surprising since transplanted neurons will be subject to a wide variety of insults, from hypoxia to mechanical trauma, free radical production, growth factor deprivation and amino acid excitotoxicity.

In previous studies from this laboratory we have shown that a variety of neurotransmitter agonists elevated intracellular Ca^2+^ ([Ca^2+^]_i_) in tyrosine hydroxylase positive (TH^+^) derived from mouse embryonic stem cells [20], [21], [22]. As these cultures develop there is an increase in post-synaptic inhibitory (presumably GABAergic) currents [20], probably due to the GABAergic neuronal population that develops alongside the TH^+^ population [21], [23]. The implication of these findings is that neuronal maturity develops over time; however there is a dearth of knowledge on the developing cell's functional capabilities as it matures. Slowly developing maturity could represent a survival challenge for transplanted cells since, during maturation, neuronal responses to GABA undergo a fundamental change; immature neurons will depolarise in response to GABA as a result of relatively high levels of [Cl^−^]_i_ [24]. Although excitatory amino acid neural toxicity is often linked to L-glutamate [25], in functionally immature neurons GABA may also be an excitatory neurotransmitter. In this study we examine, using live-cell calcium and chloride imaging, the function of ESC-derived TH^+^ and TH^+^/Pitx3^+^ (midbrain dopaminergic) neurons as they develop in monolayer culture. Our data show that the population of neurons that express TH show elevated [Cl^−^]_i_ and significant depolarization in response to GABA until around day 21 of differentiation, almost eight days after TH was first expressed. Using a post-mitotic midbrain neuron marker, Pitx3, we have also shown that three hours of incubation with GABA is enough to compromise membrane integrity, but only early in neuronal development. This study shows that the state of functional maturity may play a key role in determining the ability of cell populations to survive transplantation.

## Methods

### Neural *in vitro* differentiation of ES cells

Pluripotent E14Tg2a wild type or Pt4-1, *Pitx3*-eGFP reporter mouse ESCs were routinely cultured in maintenance media: DMEM supplemented with 10% fetal bovine serum, 0.1 mM 2-mercaptoethanol, and 2 mM GlutaMAX™-I (Invitrogen, Australia) and 2000 U mL^−1^ recombinant mouse leukemia inhibitory factor (Millipore, Australia).

Monolayer dopaminergic differentiation was initiated by seeding 3.5×10^3^ cells cm^−2^ in maintenance media and incubating cultures at 37°C in a humidified 5% CO_2_-in-air atmosphere for at least 24 h before the maintenance medium was replaced with N2B27 medium, a 1∶1 mixture of DMEM/F-12 supplemented with N2 additives, 50 µg mL^−1^ bovine albumin fraction V (Invitrogen, Australia), 25 µg mL^−1^ insulin (Sigma-Aldrich, USA) and Neurobasal media supplemented with B-27 serum-free additive (Invitrogen, Australia), and cultures maintained for 5 days. At this stage (day 5 of differentiation) cells were dissociated with Accutase (Invitrogen, Australia) and plated onto laminin-coated surfaces at 5×10^4^ cells cm^−2^ in N2B27 with 200 ng mL^−1^ recombinant mouse sonic hedgehog (Sigma-Aldrich, USA), 100 ng mL^−1^ recombinant mouse fibroblast growth factor 8b, 10 ng/mL recombinant mouse fibroblast growth factor basic (both R&D Systems, USA) and 10 ng/mL heparin sulfate (Sigma-Aldrich, USA). The culture media was replaced three days later with an equal volume of media and supplements. At day 11 the media was changed to N2B27 medium containing 200 µM L(+)-ascorbic acid (Merck, Australia), and 20 ng/mL recombinant glial derived neurotrophic factor (Sigma-Aldrich, USA). Cultures were maintained this was up to day 23, with media changes performed daily.

### Live cell [Ca^2+^]_i_ and [Cl^−^]_i_ imaging

At days 13,15,17,19 and 21 (some studies were extended to day 23) cells were incubated with fluo-4 AM (10 µM; Invitrogen, Australia) for 45 min at 37°C, washed once and incubated with HEPES buffer (of composition, in mM, NaCl 145; MgSO_4_ 1; KCl 5; glucose 10; CaCl_2_ 2.5; HEPES 10; pH 7.4) containing tetrodotoxin citrate (TTX; 1 µM; Tocris Bioscience, UK) and bovine serum albumin (0.3% w/v) and placed on a heated (37°C) stage. Cells were viewed using a Nikon TE2000U microscope coupled to a SPOT RT camera. Fluo-4 was excited at 482/35 nm using a Sutter Instruments DG-4 light box; emission was recorded at 536/40 nm. Regions were drawn around cell bodies and time-lapse images recorded at 0.33 or 1 s intervals. Background light was subtracted from each region and emission intensity calculated using Metafluor (Universal Imaging, USA). After a 10-min equilibration one of the agonists, either adenosine triphosphate (ATP, 300 µM), noradrenaline (NA, 30 µM), acetylcholine (ACh, 30 µM), L-glutamate (Glut, 30 µM), or γ-aminobutyric acid (GABA, 30 µM) was added to the culture well and the cells left for 60 s. Agonist concentrations were based on previous work from this laboratory [20], [21], [22], [23]. Cells were then washed with fresh pre-warmed buffer thrice. At least six minutes later one of the remaining agonists was added at random and the process repeated for the remaining agonists. At the end of each experiment KCl (30 mM) was added to cells to ensure viability; only those cells that gave a response to at least one agonist and KCl were included in subsequent analyses.

In another series of fluo-4 AM experiments we examined the effects of GABA antagonists upon responses to KCl (30 mM) and Glut (30 µM) on [Ca^2+^]_i_ at day 23 of differentiation. After baseline recordings were taken, KCl or Glut were added to the cultures in the absence or presence of either vehicle, the GABA_A_ antagonist bicuculline (30 µM), or the GABA_B_ antagonist CGP-55845 (6 µM) [21].

For quantitative [Ca^2+^]_i_ imaging, cells were incubated with the ratiometric Ca^2+^ fluorophore, fura-2 AM (10 µM, as described previously [26]). Briefly, cells were illuminated at 340/26 and 387/11 nm using a Sutter DG4, emissions 510/84 nm were captured and analyzed as described above. Following incubation cells were washed in HEPES buffer and subsequently incubated with KCl (30 mM). [Ca^2+^]_i_ was calculated using the equation
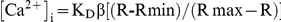

[27] where β is the emission ratio (Rmin/Rmax at 380 nm). The dissociation constant (K_D_) value of 285 nM was taken from [28]. The Rmin value was obtained in the absence of Ca^2+^ and in the presence of both the calcium ionophore 4-Br A23187 (20 µM) and EGTA (1 mM). The Rmax was obtained in the presence of both 4-Br A23187 (20 µM) and Ca^2+^ (10 mM).

For calculating [Cl^−^]_i_ we incubated neurons with the Cl^−^ fluorophore dihydro-MEQ (10 µM) [29] in normal HEPES buffer, measuring fluorescence intensity following excitation at 340/26 nm; emission was recorded at 510/84 nm. Resting state fluorescence and fluorescence following the addition of GABA (30 µM) were recorded. We changed to a Cl^−^ free buffer (normal HEPES with gluconate ions replacing Cl^−^) containing nigericin (10 µM), tributyltin (10 µM), and valinomycin (5 µM) to measure minimum fluorescence. 10 minutes later Cl^−^ was added back into the tissue buffer at 0.1, 1, 10, and 100 mM, allowing a Stern-Volmer relationship to be constructed [30] and mM [Cl^−^]_i_ to be interpolated.

Following every experiment cells were fixed with 4% (w/v) paraformaldehyde in PBS for 25 min at room temperature and then stored in kryofix (ethanol ∶ H_2_0 ∶ PEG 300; 7.9∶7.4∶1) at 4°C until immunocytochemistry was performed.

### Immunocytochemistry and eGFP visualization

Cells were washed free of kryofix (3×5 min in PBS) and permeabilized with 0.1% Triton X-100 in PBS for 30 min at room temperature, blocked with 1% normal donkey serum in PBS for 30 minutes and incubated overnight in 0.1% Triton X-100 in PBS with primary antibodies to tyrosine hydroxylase (TH; rabbit IgG, 1∶200, Chemicon, Australia) and β3-tubulin (mouse IgG, 1∶1000, Chemicon, Australia). The antigen was visualized using the secondary antibodies donkey anti-mouse Alexa Fluor® 488 and donkey anti-rabbit Alexa Fluor® 594 (both Molecular Probes, USA) at 1∶1000 for 2 h at room temperature. Cells were viewed using a Nikon TE2000U microscope coupled to a SPOT RT camera, or a Nikon A1R confocal microscope. FITC and Texas-red X filter sets were used to generate excitation and emission wavelengths. For visualization of eGFP, cells were imaged within two days of permeabilization to ensure an adequate signal could still be obtained. Fields of view corresponding with those used for Ca^2+^ and Cl^−^ imaging studies were identified by aligning initial bright field images with those taken after immunolabeling. TH immunoreactive cells (TH^+^), and later TH^+^ and Pitx3-eGFP immunoreactive cells (TH^+^GFP^+^), were identified and cell regions defined so that [Ca^2+^]_i_ and [Cl^−^]_i_ responses in those cells could be determined after replaying the initial live cell experiments.

### Cell Death experiments

On days 15 and 23 of differentiation Pt4-1 (Pitx3-eGFP) cells were incubated on a heated stage (37°C) with the nuclear dye TOPRO-3 (1 µM) and the GABA reuptake inhibitor nipecotic acid (6 µM). 10 minutes later cells were imaged using a Nikon A1R confocal microscope to detect eGFP and TOPRO-3 staining. Cells were then treated with either vehicle, GABA (30 µM), Glut (30 µM), or both GABA and Glut, and incubated at 37°C. At 1, 2, and 3 hours, cells were re-imaged for eGFP and TOPRO-3. Overlapping binary layers were added after thresholding with Nikon Elements AR (Nikon, USA) software, and cell numbers were then counted.

### Statistics

All analysis and graph fitting was performed with the graphics and statistics program PRISM v5.0 (GraphPad Software Inc., USA). Agonist or KCl-induced changes in [Ca^2+^]_i_ were determined by identifying responses that exceeded 10 standard deviations above average fluorescence intensity in the 30 seconds prior to agonist or vehicle addition. Statistical comparisons were then performed on the cells deemed to have responded to an agonist or KCl, and on the magnitude of that response. Comparison of these responses across different days of differentiation consisted of one-way ANOVA followed by a post-hoc Dunnett's test. [Ca^2+^]_i_ and [Cl^−^]_i_ quantitation was compared using one-way ANOVA followed by a post-hoc Dunnett's test. Paired t-tests were used to assess changes in [Cl^−^]_i_ following the addition of GABA (30 µM).

Neurons showing rhythmic elevations of intracellular Ca^2+^ were identified as spontaneous oscillators if the maximum responses were equal to or greater than 175% of the smallest fluorescence measurement and at least six of these oscillations, (having maxima within 10% of the largest value) were present and evident at reasonably regular time intervals. One-way ANOVA was used to analyze changes across treatment groups at different time points in cell death experiments. In all cases P<0.05 was taken as the level of significance.

### Drugs & Solutions

Acetylcholine, γ-aminobutyric acid, L-glutamate, nipecotic acid, 4-Br-A23187, bicuculline methiodide and noradrenaline were from Sigma-Aldrich (Australia). Tetrodotoxin citrate and CPG-55845 were from Tocris Bioscience (UK). Fura-2 AM, MEQ, fluo-4 AM, TOPRO-3 iodide, nigericin, tributyltin and valinomycin were from Invitrogen (Australia). dihydro-MEQ was prepared fresh daily from MEQ powder according to the manufacturer's directions. Noradrenaline was diluted in buffer containing ascorbic acid (100 µM).

## Results

### Immunocytochemistry and Fluo-4 calcium imaging

**Figure 1:**
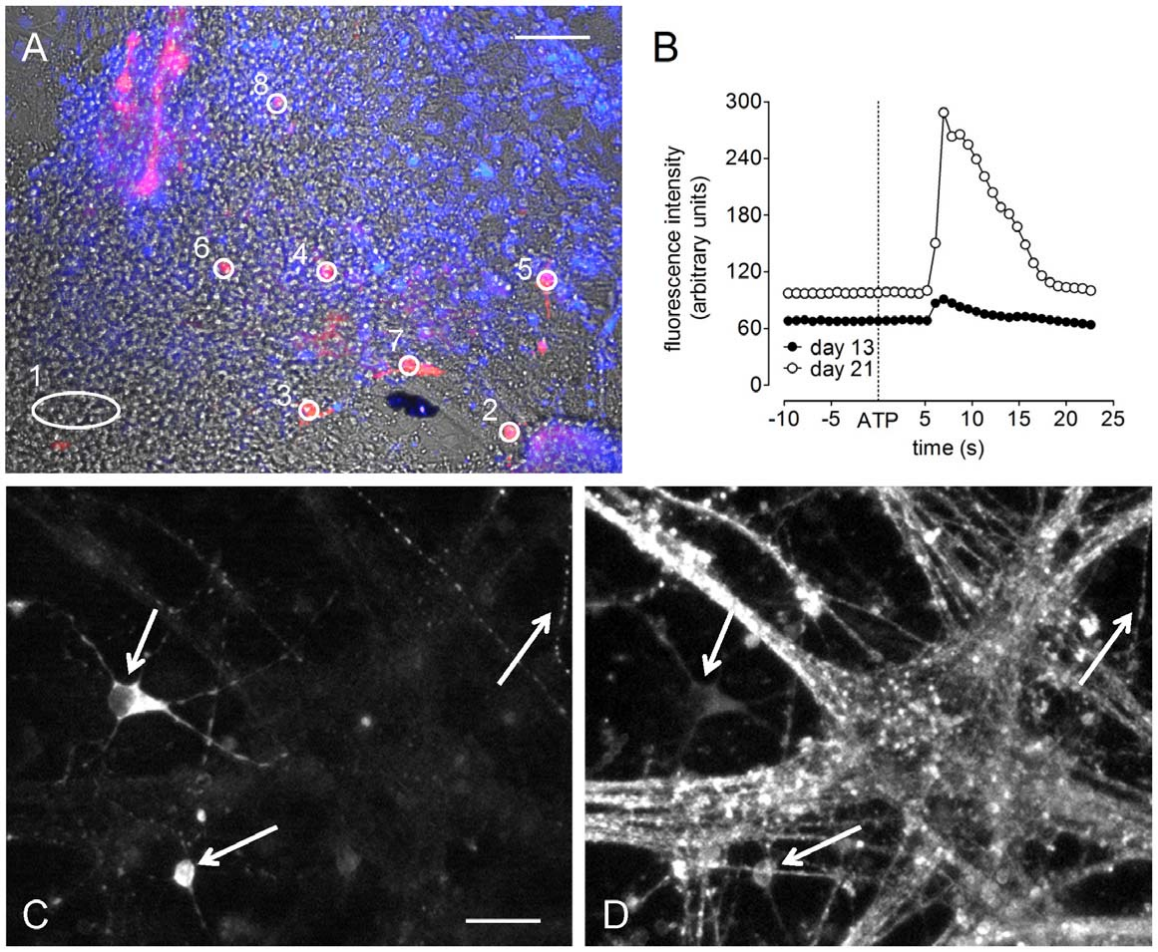
Calcium imaging methods and immunocytochemical characterization of neurons. Cultures were loaded with the calcium sensitive fluorophore, fluo-4 AM (10 µM; blue overlay on 10× bright field image in (**A**)). Drugs were added to culture and fluorescent recordings taken. Post experiment immunocytochemistry allowed for TH^+^ cells to be identified (red overlay in (**A**)). Regions were drawn within cells enabling fluorescence intensity to be measured after replaying the initial experiment (regions 2–8 are TH^+^ cells and region 1 is for the subtraction of background fluorescence). TH^+^ cells in mounds were generally larger, and excluded from analysis (see (**A**), top left corner). A typical fluorescent response after application of ATP (300 µM) at day 13, and at day 23, is shown in (**B**). (**C**) shows TH^+^ immunoreactivity and (**D**) shows β3-tubulin^+^ immunoreactivity for the same field of view at day 21. Note the different morphologies of the TH^+^ cells (left most arrows), as well as TH/β3-tubulin^+^ varicosities (right arrow). Scale bars; (**A**) 100 µm, (**C and D**) 50 µm.

From day seven of differentiation large colonies of cells developed. TH^+^ cells were consistently visible in and around the periphery of these colonies from day 13 of differentiation. Figure 1A shows a bright field image of cells at day 21, with TH^+^ cells overlaid in red and cells loaded with fluo-4 in blue. Consistent with a previous study from this laboratory [22], fluo-4 AM loaded mouse ESC-derived TH^+^ neurons responded to ATP (300 µM), NA (30 µM), ACh (30 µM), Glut (30 µM) and KCl (30 mM) with elevations of [Ca^2+^]_i_. Figure 1B shows typical TH^+^ neuron responses to ATP (300 µM) at days 13 and 23 of differentiation. Figures 1C and 1D show that typical TH immunoreactive neurons were also immunoreactive to the pan-neuronal marker protein, β3-tubulin.

**Figure 2:**
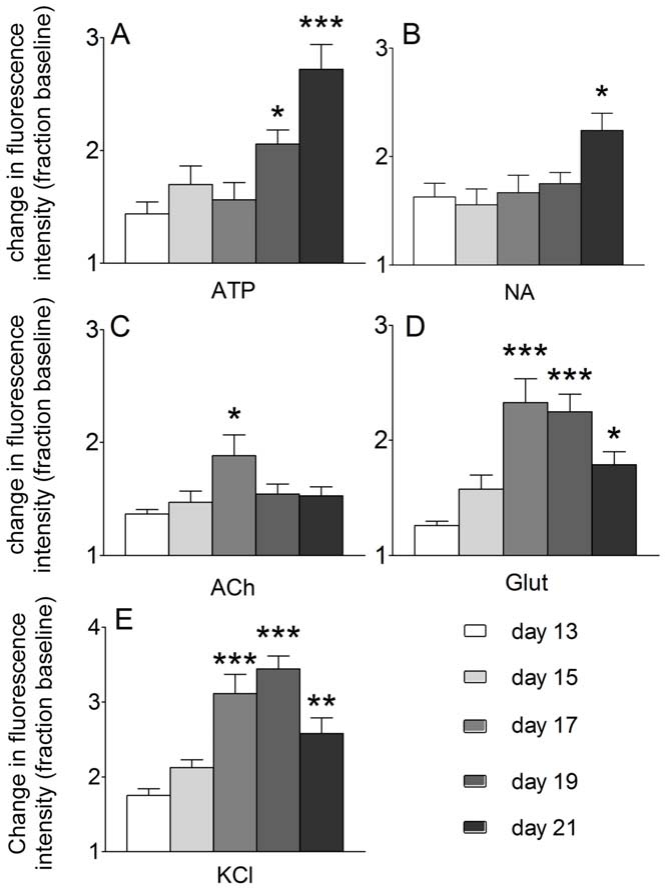
Calcium responses develop during the differentiation of TH^+^ neurons. Changes in [Ca^2+^]_i_ were recorded after stimulation with ATP 300 µM (**A**), NA 30 µM (**B**), ACh 30 µM (**C**), Glut 30 µM (**D**), and KCl 30 mM (**E**). Cells did not respond to vehicle (data not shown). The agonist-induced maximal fluorescence intensity generally increased beyond day 15 of differentiation (P<0.05, one way ANOVA with post-hoc Dunnett's test comparing to day 13, n = 4–6, columns show mean maximal responses ± SEM).

ATP elicited modest elevations of [Ca^2+^]_i_ in TH^+^ neurons at days 13,15 and 17 of differentiation, by day 19 the responses to ATP were significantly larger than at day 13 (P<0.05 & 0.001, one-way ANOVA, post-hoc Dunnett's multiple comparison test, n = 4–6; Figure 2A). Responses to NA were also elevated, but at day 21 only (P<0.05, one-way ANOVA, post-hoc Dunnett's multiple comparison test, n = 4–6; Figure 2B). Responses to both ACh and Glut were elevated on day 17 of differentiation, and both declined thereafter (P<0.05 & 0.001, one-way ANOVA, post-hoc Dunnett's multiple comparison test, n = 4–6; Figure 2C and 2D). Compared to day 13 TH^+^ cells, depolarization responses (KCl, 30 mM) were elevated at days 17, 19 and 21 (P<0.01, one-way ANOVA, post-hoc Dunnett's multiple comparison test, n = 4–6; Figure 2E).

### [Ca^2+^]_i_ and [Cl^−^]_i_ measurements in Fura-2 and MEQ loaded TH^+^ cells

To examine the possibility that changes in agonist responsiveness were due to changes in neuronal maturity, we calculated resting [Ca^2+^]_i_ and [Cl^−^]_i_ in Fura-2 and MEQ loaded cultures across differentiation.

**Figure 3:**
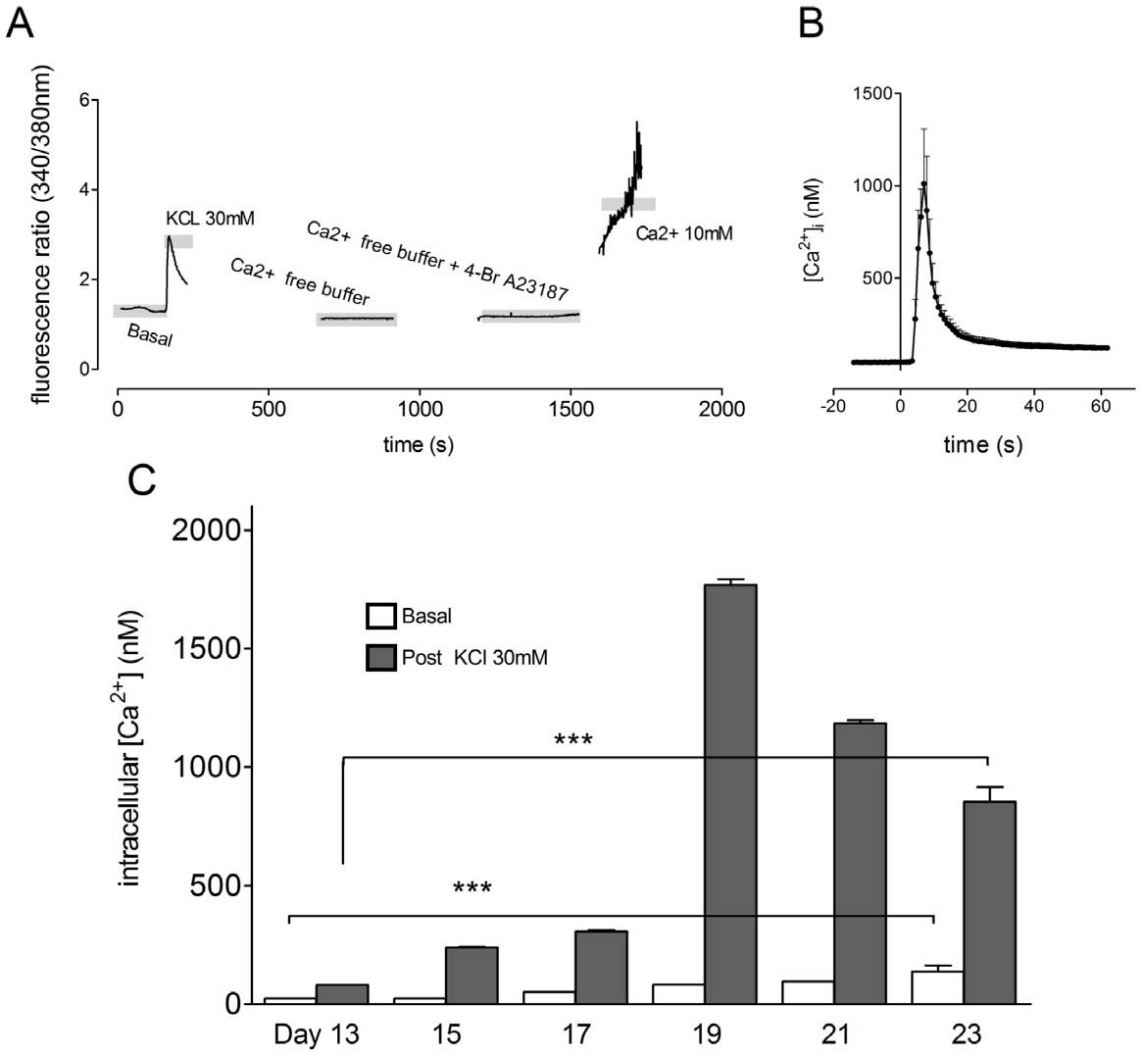
[Ca^2+^]_i_ quantitation during differentiation. Cells were loaded with the ratiometric calcium indicator Fura-2AM and [Ca^2+^]_i_ quantitated. (**A**) shows a typical trace of Ca^2+^ related fluorescence over time. Initially basal and KCl (30 mM)-stimulated Ca^2+^ related fluorescence were calculated. The experimental buffer was then changed to a calcium free HEPES buffer containing EGTA (1 mM) and the Ca^2+^ ionophore, 4Br-A23187 (20 µM); excess Ca^2+^ (10 mM) was added later. Combined, these conditions allowed for the calculation of [Ca^2+^]_i_ as described in the main text. (**B**) shows mean ± SEM basal and KCl-stimulated [Ca^2+^]_i_ for all TH+ neurons in a single field of view (8 neurons; KCl was added at time = 0 sec). (**C**) Both basal and post-KCl [Ca^2+^]_i_ increased significantly during differentiation (; one way ANOVA with post-hoc Dunnett's test, P<0.05, n = 4–6).

Figure 3A shows a typical trace of Ca^2+^ related fluorescence in a single TH^+^ neuron at day 21 of differentiation. [Ca^2+^]_i_ then could be calculated (as described in Methods) using the measurements from such traces. Figure 3B shows the mean (+upper 95% confidence interval) response to KCl (30 mM) of ten TH^+^ cells from one field of view (n = 1), expressed as change in nM [Ca^2+^]_i_. Mean basal [Ca^2+^]_i_ increased between days 13 and 21, from 24.6 (95% confidence intervals 24.3; 25.0) nM to 94.6 (93.8; 95.4) nM (Figure 3C, n = 4–6). Across the same time period the maximal [Ca^2+^]_i_ elevation induced by KCl (30 mM) increased from 81.1 (79.9; 82.3) nM to a peak of 1.77 (1.74; 1.79) µM at day 19 (Figure 3C, n = 4–6).

**Figure 4:**
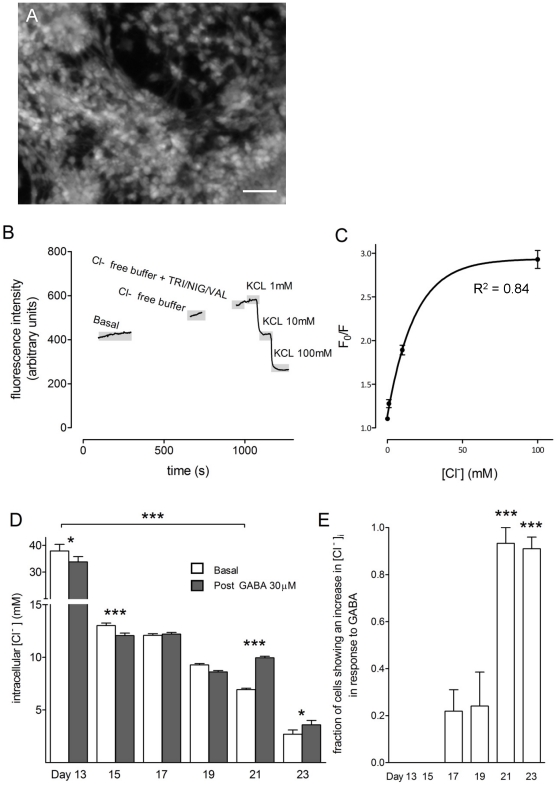
Intracellular chloride in TH^+^ neurons over time. Cells were loaded with the chloride indicator dihydro-MEQ and illuminated at 340 nm, (**A**) shows a typical field of view. Basal [Cl^−^] was recorded (and in some cases responses to GABA, 30 µM, not shown in this trace). The addition of the ionophores nigericin (10 µM), tributyltin (10 µM), and valinomycin (5 µM) enabled a standard curve to be constucted, (**C**), where F_0_/F is the ratio of fluorescence in Cl− free buffer containing the ionophores over fluorescence intensities at various [Cl^−^]. Basal and post-GABA [Cl^−^]_i_ were interpolated from the standard curve. Basal [Cl^−^]_i_ decreased from day 13 to day 23 of differentiation ((**D**); one way ANOVA with post-hoc Dunnett's test, P<0.05, n = 4–6). This panel also shows that, at days 13 and 15 GABA (30 µM) reduced [Cl^−^]_i_, but by day 21 elevated [Cl^−^]_i_ (Student's paired t-test, P<0.05, n = 4–6). The fraction of cells to have elevated [Cl^−^]_i_ in response to GABA increased significantly over differentiation ((**E**); one way ANOVA with post-hoc Dunnett's test, P<0.05, n = 4–6).

Figure 4A shows a typical MEQ fluorescence field of view. The experimental conditions enabling us to calculate [Cl^−^]_i_ are shown in figure 4B (MEQ fluorescence intensity is *inversely* proportional to [Cl^−^]_i_,). This inverse relationship led to the creation of standard curves (one-phase association, R^2^ value of 0.84) known as Stern-Volmer plots (figure 4C) from which [Cl^−^]_i_ was interpolated. Between days 13 and 21 [Cl^−^]_i_ decreased from 56.0 (55.1; 56.9) to 6.9 (6.8; 7.1) mM (Figure 4D; one way ANOVA with post-hoc Dunnett's test, P<0.001, n = 3–4). Only by day 21 did GABA (30 µM) elicit a significant elevation of [Cl^−^]_i_ (Figure 4D; Student's paired t-test, P<0.05 and 0.001). Before this time point, GABA either did not affect resting [Cl^−^]_i_ or reduced it (P<0.05 and 0.001). The percentage of the TH^+^ population in which GABA elevated [Cl^−^]_i_ increased from 0% at day 13, to 91±5% at day 23 (Figure 4E; P<0.001, n = 3–4).

### Responses to GABA in Fluo-4 loaded TH^+^ cells

**Figure 5:**
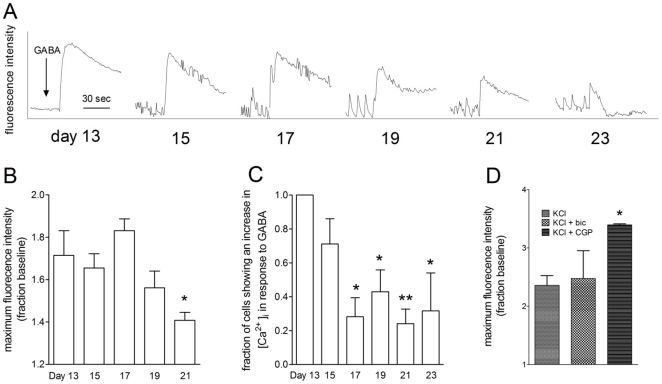
Responses to GABA during differentiation. GABA elevated [Ca^2+^]_i_ in TH^+^ cells, especially early in differentiation. (**A**) shows traces of the GABA response declining from days 13 to 23. The mean magnitude of the maximal responses to GABA decreased over the course of differentiation (**B**), as did the number of cells which were able to produce an elevation of [Ca^2+^]_i_ ((**C**); one way ANOVA with post-hoc Dunnett's test, P<0.05, n = 4). The GABA_B_ antagonist CGP-55845 (6 µM), but not the GABA_A_ antagonist bicuculline (10 µM) increased (one-way ANOVA, post-hoc Dunnett's multiple comparison test, n = 4) the response to KCl at day 21 of differentiation (**D**).

GABA (30 µM) elicited robust elevations of [Ca^2+^]_i_ at day 13 of differentiation, see Figure 5A for typical traces. During differentiation these response progressively decreased (P<0.05, one-way ANOVA with post-hoc Dunnett's multiple comparison test, n = 4, Figure 5B). In addition the number of TH^+^ cells responding to GABA (30 µM) with elevations of [Ca^2+^]_i_ decreased from 100% at day 13 to around 25% by day 21 (P<0.05, one-way ANOVA, post-hoc Dunnett's multiple comparison test, n = 4, Figure 5C). At day 17 of differentiation the GABA- (30 µM) mediated elevation of [Ca^2+^]_i_ was completely blocked by the GABA_A_ antagonist, bicuculline (10 µM), but not by the GABA_B_ antagonist, CGP-55845 (6 µM; data not shown). To assess the effects of endogenous GABA at later days of differentiation we incubated neurons with CGP-55845 (6 µM) and bicuculline (10 µM). CGP-55845 significantly increased the KCl-mediated elevation of [Ca^2+^]_i_ at day 21 of differentiation (P<0.05, one-way ANOVA, post-hoc Dunnett's multiple comparison test, n = 4, Figure 5D). This was also true of the Glut-mediated elevation of [Ca^2+^]_i_ (data not shown).

**Figure 6:**
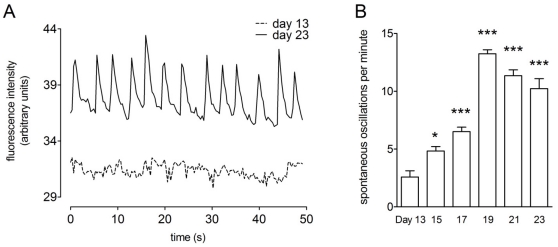
Spontaneous calcium oscillations in TH^+^ neurons during differentiation. (**A**) typical traces showing the regular spontaneous oscillations of [Ca^2+^]_i_ evident at day 23, but not at day 13 of differentiation. (**B**) shows the mean ± SEM of spontaneous oscillations observed per minute during differentiation (one way ANOVA with post-hoc Dunnett's test, P<0.05, n = 4).

During differentiation we noticed a significant increase in the frequency of spontaneous calcium oscillations in TH^+^ cells (one-way ANOVA, post-hoc Dunnett's test, P<0.01, n = 4; Figure 6A shows typical spontaneous oscillations and 6B shows the mean (± SEM) frequency of these events).

### Pitx3-eGFP cell imaging in Fura-2 and MEQ loaded cells

**Figure 7:**
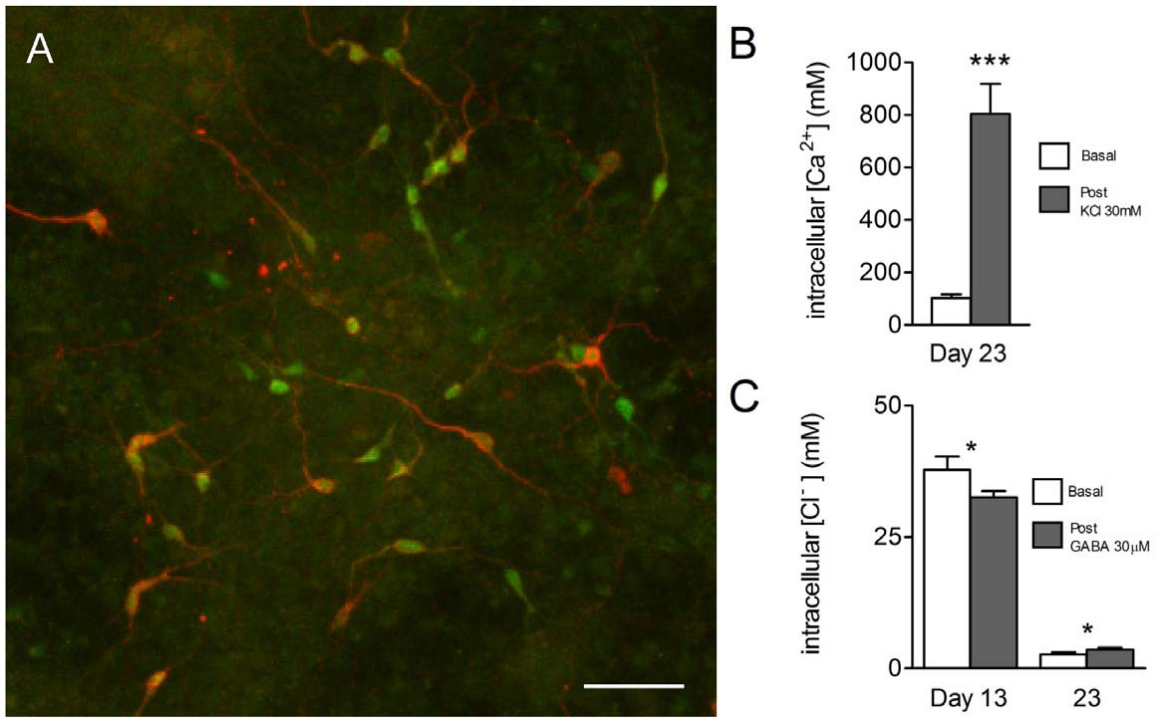
Characterisation of [Ca^2+^]_i_ and [Cl^−^]_i_ in *Pitx3*-eGFP^+^ cells. eGFP was highly co-localised with TH at all days; (**A**) shows a day 23 culture of TH^+^GFP^+^ cells. (**B**) Basal [Ca^2+^]_i_ was 103 (91,115) nM at day 23. KCl (30 mM) elevated [Ca^2+^]_i_ to 806 (716,919) nM (P<0.001, Student's unpaired t-test, n = 4). (**C**) Basal [Cl^−^]_i_ fell from 38 (36, 40) mM at day 13 to 2.7 (2.4,3.0) mM at day 23. GABA (30 µM) caused a significant reduction in [Cl^−^]_i_ at day 13 (Student's paired t-test, P<0.05, n = 4) and a significant increase in [Cl^−^]_i_ at day 23 (Student's paired t-test, P<0.05, n = 4).

We next measured [Ca^2+^]_i_ and [Cl^−^]_i_ in a Pitx3-eGFP ESC line [31] to establish whether the expression of Pitx3 correlated with cellular maturity. eGFP was seen from days 13 to 23 and was highly co-localised with TH immunoreactivity (Figure 7A). Consistent with the earlier experiments in TH^+^ only cells, resting [Ca^2+^]_i_ in TH^+^GFP^+^ cells at day 23 was 103 (91,115) nM, this rose to 806 (716,919) nM after KCl (30 mM, Figure 7B). Resting [Cl^−^]_i_ fell from 38 (36, 40) mM at day 13 to 2.7 (2.4, 3.0) mM at day 23 (Figure 7C). GABA (30 µM) caused a significant reduction in [Cl^−^]_i_ at day 13 (Student's paired t-test, P<0.05; n = 4, Figure 7C) and a significant elevation in [Cl^−^]_i_ at day 23 (Student's paired t-test, P<0.05; n = 4, Figure 7C).

### Pitx3-eGFP cell death in TOPRO-3 loaded cells

**Figure 8:**
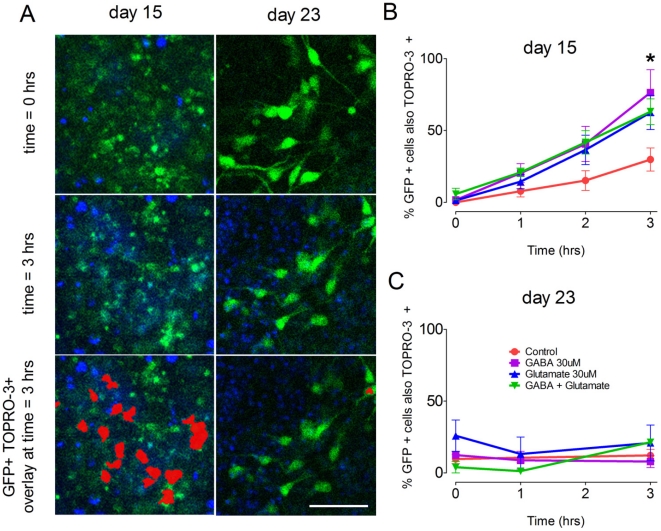
Exposure to amino acid neurotransmitters causes cell death in early GFP^+^ cultures. Cultures containing Pitx3-eGFP^+^ cells were incubated with the membrane impermeable dye TOPRO-3 and exposed to GABA (30 µM), Glut (30 µM), or a combination of the two. (**A**) shows the effects of GABA exposure on cells at day 15 and day 23. The panels show, from top to bottom, eGFP and TOPRO-3 at time = 0, after 3 hours of constant incubation, and overlays of regions showing increased TOPRO-3 reactivity. After 3 hours of amino acid incubation there were a significantly higher number of red overlays in non-vehicle control treated cells from day 15 (One way ANOVA with post-hoc Dunnett's test, P<0.05, n = 5) (**B**) than were present in cultures from day 23 (**C**).

Finally, we investigated the possibility that the excitation of neurons early in differentiation would cause cell death. At day 15, a 3 hour incubation of cells with GABA (30 µM), Glut (30 µM), or GABA and Glut in combination (both 30 µM) increased (P<0.05; one-way ANOVA with post-hoc Dunnett's test, n = 5) the number of TOPRO-3^+^ eGFP^+^ cells (Figure 8A shows typical fields of view at days 15 and 23). Figure 8B shows mean (± SEM) number of TOPRO-3^+^ eGFP^+^ cells, expressed as a percentage of the original number of eGFP^+^ cells. Figure 8C shows that by day 23 incubation with GABA or Glut did not increase TOPRO-3^+^ labeling (n = 5).

## Discussion

We have investigated the development of functional maturity in tyrosine hydroxylase, and Pitx3-eGFP expressing neurons during differentiation. Immunocytochemistry confirmed the presence of TH and β3-tubulin consistently from day 13 of differentiation, indicating that, under these conditions, the cell populations contain catecholaminergic neurons. Even at day 13, the majority of TH^+^ cells responded to the agonists ATP, NA, ACh and Glut with elevations of [Ca^2+^]_i_. Although the number of responding neurons was consistently close to 100% across the timeframe of this study (data not shown), the magnitude of the responses was varied. ATP and NA responses increased in magnitude across days 13 to 21, but both ACh and Glut peaked at day 17 and declined thereafter. KCl responses peaked at day 19 and declined by day 21. That we saw such variety in the magnitude of agonist responses in our cultures is perhaps consistent with the idea of developing cell culture maturity. These cultures contain a large population of GABAergic neurons [21] which may explain why the [Ca^2+^]_i_ response to depolarization by KCl was considerably elevated by the addition of the GABA_B_ receptor antagonist, CGP-55845 at day 21 of differentiation. This too is consistent with the development of inhibitory post-synaptic potentials [20]. Thus, as the cultures develop, it appears that endogenous GABA may begin to exert an inhibitory effect on TH^+^ neurons.

Soon after the appearance of detectable TH^+^ these neurons responded to GABA with elevations of [Ca^2+^]_i_, in contrast to the responses to other agonists, both the magnitude of response, and the number of cells responding, consistently decreased from days 13 to 23. This finding is broadly consistent with data showing that, in the developing rat mesencephalon, amino acid transmitters hyperpolarize neurons at E13, but begin to depolarize from E15 [32]. One of the key indicators of neuronal maturity is the concentration of intracellular Cl^−^, which falls as neurons mature (for a comprehensive review see Ben Ari et al. [24]). We therefore assessed basal [Ca^2+^]_i_ and [Cl^−^]_i_ at six time points within our monolayer differentiation paradigm. To our knowledge this is the first time that [Cl^−^]_i_ has been measured in a longitudinal fashion in ESC derived TH+ neurons. [Cl^−^]_i_ fell between days 13 and 23 and, more importantly, the addition of GABA did not elevate [Cl^−^]_i_ in the majority of cells until day 21, indicating that the majority of cells were functionally immature up until, or just before this time. Cordero-Erausquin and co-workers [33] demonstrated that GABA elicits both increases and decreases in [Ca^2+^]_i_ in spinal lamina I neurons, in addition the depolarizing effect of GABA disappeared with development [34]. More recently, Pignatelli and co-workers [35] have shown that the tyrosine hydroxylase expressing cells of the glomerular layer of the olfactory bulb have a [Cl^−^]_i_ of ∼5.5 mM, and that cells lying in the external plexiform layer and the mitral cell and internal plexiform layers have [Cl^−^]_i_ of ∼9 and 10.5 mM, respectively [35]. This range of concentrations of [Cl^−^]_i_ is broadly consistent with those seen as cells matured in this study. The change from excitatory to inhibitory GABA effects is, in large part, due to the increased expression of the potassium-chloride co-transporter 2 [36], [37], [38]. As basal [Cl^−^]_i_ decreased with increasing time in culture there was a concomitant increase in basal [Ca^2+^]_i_. From day 19, the levels of resting [Ca^2+^]_i_ we report are largely consistent with reports from primary cultured (rat) post-natal Purkinje fibers [39], developing cortical neurons (E18, [40]) and motoneurons (E15, [41]). The only study to investigate time dependent effects in culture reported an elevation of resting [Ca^2+^]_i_, from 73 to 139 nM, over days three to six of culture [41]. Another indicator of cellular maturity may be the development of spontaneous activity in our cultures as [Ca^2+^]_i_ increased and [Cl^−^]_i_ decreased. Gruol and co-workers [39] reported that cells with little electrical activity had low resting [Ca^2+^]_i_ levels (∼50 nM); this may be consistent with our changes in [Ca^2+^]_i_ during differentiation. This developing spontaneous activity may also be consistent with the observation of spontaneous and burst firing potentials in dopaminergic neurons [42], [43], [44], [45]. Ca^2+^ signaling responses have not been studied widely in ESC-derived neuronal cultures before; apart from our previous work, a recent study by Malmersjo and co-workers [46] in human ESC-derived TH^+^ neurons observed the development of Ca2+ responses in those cells (to, for example, KCl, Glut, or neurotensin stimulation) and concluded there were functional similarities to primary ventral midbrain dopaminergic neurons of mice.

To further investigate the development of cellular maturity we utilized a cell line in which eGFP is expressed under the control of the Pitx3 promoter [31], this cell line allowed us to examine [Ca^2+^]_i_ and [Cl^−^]_i_ in midbrain specific dopaminergic neurons. These cells expressed Pitx3 from day 13 and, consistent with our findings in the wild type (TH^+^) neurons, showed high levels of [Cl^−^]_i_ and responded to GABA with a reduction of [Cl^−^]_i_. By day 23 when intracellular [Ca^2+^]_i_, was high, and [Cl^−^]_i_ was low, and these neurons responded to GABA with an elevation of [Cl^−^]_i_. These data indicate that the TH^+^GFP^+^ cells show similar functional development to the TH^+^ cells, and also that neuronal maturity (with respect to [Cl^−^]_i_ handling) lags behind the expression of Pitx3. These neurons also express other genetic markers typical of midbrain dopaminergic neurons; using reverse transcriptase PCR we found *Gbx2* and *Lmx1a* are present from day 3, *Nurr1*, *Th*, and *Pax2* from day 7, and *Pitx3* from day 10 (unpublished data). These developmental markers indicate that the immature functionality we see in this study takes place in the context of correctly specified, post-mitotic ‘mature’ cells.

Excitatory effects of GABA in mature systems have been identified following the activation of GABA_A_ receptors within immature neurons and in small cell compartments, for example dendrites, due to their high receptor to volume ratio [24]. This is in stark contrast with the tonic inhibitory, and largely neuroprotective role GABA plays in the adult striatum [47]. Our finding that a sustained exposure to GABA early in neuronal development (day 15) caused the cell membranes of Pitx3^+^ neurons become permeable to membrane impermeant nuclear dye, TOPRO-3, illustrates the fragility of these early neurons. We therefore suggest that immature neurons may struggle to survive in regions containing high concentrations of either or both GABA and L-glutamate. In human hippocampal brain slices GABA is present at concentrations of ∼8 µM [48]. This value is not far removed from the concentration of GABA we used for calcium imaging and the neurotoxicity assays (30 µM), giving our study physiological relevance. At present we believe that this amino acid mediated neurotoxicity may result in calcium overload and cell death, consistent with findings in other neuronal systems [49], [50], [51]. The determination of optimal day of transplant is work still to be undertaken, given that mature cells may also suffer from poor survival after their axons and neurites are severed [11]. The difficulty in transplantation will be achieving a balance between neuronal maturity and an ability to sort specific populations in preparation for transplantation before the cultures become too overburdened with dendritic and axonal processes. From our study, mouse cells may be ready for sorting and transplantation from day 20, although we have seen significant post-sorting cell survival even up to day 26 (unpublished observation). Clearly, establishing the development of human neuron functional maturity will require independent assessment based upon each differentiation paradigm.

In this study we have shown that dopaminergic neuron responses to agonists and KCl generally increase during differentiation when [Cl^−^]_i_ decreases and [Ca^2+^]_i_ increases. Of perhaps most significance is our data showing that these cultures functionally mature well after the appearance of the post-mitotic marker of midbrain dopaminergic neurons, Pitx3. We conclude that functional maturity should be taken into account in cell populations prior to transplantation since functionally immature cells may be susceptible to amino acid cytotoxicity, in spite of the expression of post-mitotic cell markers.
